# Anti-Inflammatory Activity of 1,6,7-Trihydroxy-2-(1,1-dimethyl-2-propenyl)-3-methoxyxanthone Isolated from *Cudrania tricuspidata* via NF-κB, MAPK, and HO-1 Signaling Pathways in Lipopolysaccharide-Stimulated RAW 264.7 and BV2 Cells

**DOI:** 10.3390/molecules28217299

**Published:** 2023-10-27

**Authors:** Wonmin Ko, Jong-Suep Baek, Zhiming Liu, Linsha Dong, Nayeon Kim, Hwan Lee, Chi-Su Yoon, Na Young Kim, Sam Cheol Kim, Dong-Sung Lee

**Affiliations:** 1 College of Pharmacy, Wonkwang University, 460, Iksan-daero, Iksan-si 54538, Republic of Korea; rabis815@naver.com (W.K.); ycs1991@naver.com (C.-S.Y.); 2 Department of Bio-Health Convergence, Kangwon National University, 1, Kangwondaehak-gil, Chuncheon-si 24341, Republic of Korea; jsbaek@kangwon.ac.kr; 3 College of Pharmacy, Chosun University, 309, Pilmun-daero, Dong-gu, Gwangju 61452, Republic of Korea; lzmqust@126.com (Z.L.); donglinsha011@163.com (L.D.); rlaskdus1209@naver.com (N.K.); ghksdldi123@hanmail.net (H.L.); 4 Pathology Division, National Institute of Fisheries Science, 216, Gijanghaean-ro, Gijang-eup, Gijang-gun, Busan 46083, Republic of Korea; pharm001@korea.kr; 5 Department of Family Practice and Community Medicine, Chosun University College of Medicine, 309, Pilmun-daero, Dong-gu, Gwangju 61452, Republic of Korea; schkim@chosun.ac.kr

**Keywords:** 1,6,7-Trihydroxy-2-(1,1-dimethyl-2-propenyl)-3-methoxyxanthone, mouse macrophage RAW 264.7, mouse microglia BV2

## Abstract

Neuroinflammation activated by microglia affects inflammatory pain development. This study aimed to explore the anti-inflammatory properties and mechanisms of 1,6,7-trihydroxy-2-(1,1-dimethyl-2-propenyl)-3-methoxyxanthone (THMX) from *Cudrania tricuspidata* in microglia activation-mediated inflammatory pain. In RAW 264.7 and BV2 cells, THMX has been shown to reduce lipopolysaccharide (LPS)-induced inducible nitric oxide synthase (iNOS), cyclooxygenase-2 (COX-2), and pro-inflammatory mediators and cytokines, including nitric oxide (NO), prostaglandin (PG) E2, interleukin (IL)-6, and tumor necrosis factor alpha (TNF-α). THMX also decreased LPS-induced phosphorylation of mitogen-activated protein kinase (MAPK) and the activation of p65 nuclear factor kappa B (NF-κB). Interestingly, THMX also activated heme oxygenase (HO)-1 expression. These findings suggest that THMX is a promising biologically active compound against inflammation through preventing MAPKs and NF-ĸB and activating HO-1 signaling pathways.

## 1. Introduction

By eliminating dangerous substances and restoring cells and tissues, neuroinflammation is an essential immune response that prevents against injury and infection; however, acute or chronic inflammation can cause severe illnesses, such sepsis, arthritis, Alzheimer’s disease, asthma, and inflammatory bowel disease. [1,2,3,4]. Therefore, the control of inflammation is critical for the therapy of these disorders.

NF-κB and MAPK signaling pathways are well known as major pathways in the regulation of the inflammatory response. First, NF-κB signaling is related to controlling cellular proliferation, cell adhesion, and inflammation [5]. In unstimulated situations, NF-κB comprising Rel family p50 and p65 subunits is generally restricted to an inactive cytoplasmic complex by binding to an inhibitory protein (I-κB). NF-κB activation causes the transcription of several pro-inflammatory cytokines and key enzymes related to the maintenance of inflammation, including tumor necrosis factor (TNF)-α and interleukin (IL)-6 [6]. In contrast, mitogen-activated protein kinase (MAPK) is a signaling cascade that mediates the production of inflammatory mediators in activated macrophages. The three major subfamilies of the MAPK family are c-Jun N-terminal kinase (JNK), p38 MAPK, and extracellular signal-regulated kinase (ERK) [7]. Numerous clinical illnesses, including amyotrophic lateral sclerosis, Parkinson’s disease, and some cancers, have been correlated with continuous activation of MAPK signaling pathways [8,9]. Therefore, the development of novel anti-inflammatory medicines should consider both the NF-κB and MAPK signaling pathways as dominant biological targets.

Oxidative stress also has a significant relation with inflammation because it is one of numerous stimuli that can cause inflammatory responses in a host. Heme oxygenase (HO)-1 is a powerful antioxidant that has a cellular protective effect in pathophysiological conditions such as cerebral ischemic injury and hypoxia [10,11,12]. Heme is degraded into biliverdin, carbon monoxide (CM), and free iron by the rate-limiting enzyme HO-1. Cyclooxygenase-2 (COX-2), inducible nitric oxide synthase (iNOS), and inflammatory cytokines are produced less when HO-1 and its byproduct, CM, are present. This reduces the levels of iNOS-derived nitric oxide (NO) and COX-2-derived prostaglandin (PG) E2 [13]. Thus, this increased expression of HO-1 results in the anti-neuroinflammatory effect of many drugs [14,15,16,17].

*Cudrania tricuspidata* is commonly found in East Asia and is classified under the Moraceae family [18]. This deciduous plant is typically found in dense shrubbery and may be found in lowlands, foothills, forests, and altitudes between 500 and 2000 m. Although it can eventually reach a height of around 1.0 to 7.0 m, it is typically seen as thickets or tiny trees [19]. *C. tricuspidata* is a plant that has been used in traditional Chinese medicine (TCM) to treat bruising, lumbago, and stomach discomfort. It has also been utilized in the form of syrup, granules, or injections [19]. In addition, the stems and roots of *C. tricuspidata* have also been used for a long time in China to make herbal teas and other useful functional beverages [19]. In South Korea, the edible fruits of *C. tricuspidata* have been used to make jam, alcoholic beverages, juice, health supplements, and other functional health products [20]. The development of this fruit as a component in dietary supplements and functional foods is increasing across multiple areas as a result of its helpful functions, such as its antioxidant, anti-inflammatory, and immune-modulating activities [20]. *C. tricuspidata* contains a variety of compounds belonging to different chemical structural categories, such as xanthones, flavonoids, organic acids, polysaccharides, phenylpropanoids, and other ingredients [19]. The main chemical skeletons identified in *C. tricuspidata* are xanthones and flavonoids, with these compounds exhibiting important pharmacological activities, such as anti-inflammatory, antioxidant, and anti-tumor effects [19]. It has been reported that over 100 xanthone compounds have been isolated, and more than 150 types of flavonoids, which can be classified into flavones, flavanones, and isoflavones, are known to be present in *C. tricuspidata* [19]. Numerous studies have reported that extracts and compounds derived from *C. tricuspidata* exhibit anti-inflammatory properties, and they are known to have anti-inflammatory molecular mechanisms that could be elucidated on the well-known signaling pathways, such as NF-κB, JAKS/ STAT, and MAPKs [19]. Furthermore, it has been reported that the fruit extract of *C. tricuspidata* exhibits neuroprotective effects against scopolamine-induced neuronal damage by regulating the CREB and ERK1/2 signaling pathways [21]. It has also been reported that the root extracts of *C. tricuspidata* decreased methylglyoxal-induced oxidative stress and inflammation by blocking PKC activation and NOX4 expression, as well as enhancing the Nrf2-antioxidant enzyme pathway in HK-2 cells [22]. In addition to this, diverse research results have also reported various other effects, including anti-tumor, hepatoprotective, anti-obesity, and immunomodulatory, as well as skin inflammation inhibition and protection effects [23,24,25,26,27].

In our prior study, 16 prenylated flavonoids and prenylated xanthones were investigated during a chemical investigation of *C. tricuspidata* [18]. Among the 16 compounds in the previous study, 1,6,7-trihydroxy-2-(1,1-dimethyl-2-propenyl)-3-methoxyxanthone (THMX) showed PTP1B inhibitory effects. In addition, in our other studies, we also confirmed that it has an inhibitory effect on skin inflammation in TNF-α/INF-γ-induced HaCaT keratinocyte [28]; however, the anti-neuroinflammatory effects and mechanism of action of THMX have not yet been reported. Therefore, THMX was further examined in the current study in the LPS-challenged RAW264.7 macrophage and BV2 brain microglia.

## 2. Results

### 2.1. Effects of THMX on the Viability of RAW264.7 and BV2 Cells

**Figure 1:**
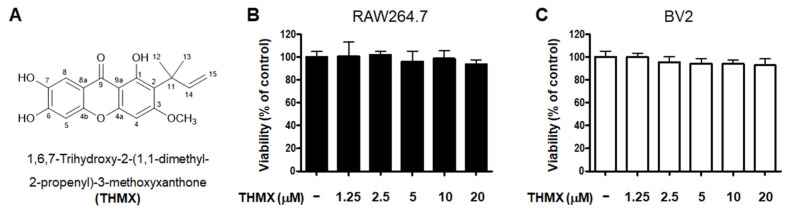
Chemical structure of THMX (**A**) and its effects on cell viability. RAW264.7 (**B**) and BV2 (**C**) cells were incubated for 24 h with various concentrations of THMX. Cell viability was determined using 3-(4,5-dimethylthiazol-2-yl)-2,5-diphenyltetrazolium bromide assays. Bars represent the means ± standard deviation of three independent experiments.

THMX was obtained from the roots of *C. tricuspidata* using a previously established isolation method [18]. The chemical structure of this compound has been previously described [18]. The cytotoxic effects of THMX were tested by treating RAW264.7 and BV2 cells with the indicated concentrations of THMX for 24 h and then performing a 3-(4,5-dimethylthiazol-2-yl)-2,5-diphenyltetrazolium bromide (MTT) assay (Figure 1). The results indicate that the safe concentration range is 1.25–20 μM.

### 2.2. Effects of THMX on Pro-Inflammatory Factors in RAW 264.7 and BV2 Cells

**Figure 2:**
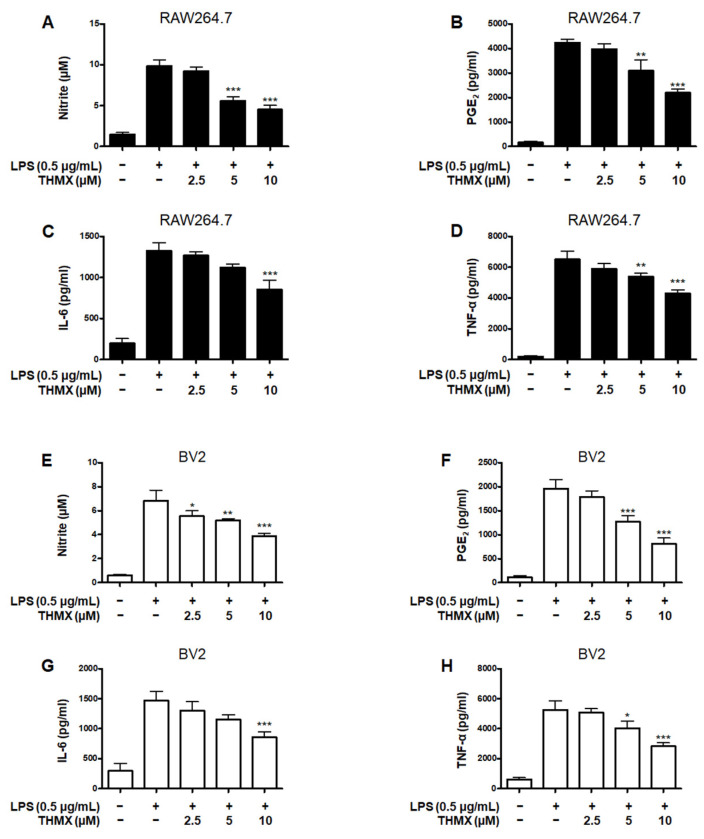
Effects of THMX on nitrite (**A**,**E**) contents and prostaglandin E_2_ (PGE_2_) (**B**,**F**), interleukin (IL)-6 (**C**,**G**), and tumor necrosis factor (TNF)-α (**D**,**H**) levels in lipopolysaccharide (LPS)-induced RAW 264.7 and BV2 cells. Cells were pretreated for 2 h with THMX and stimulated for 24 h with LPS (0.5 μg/mL). Bars represent the means ± standard deviation of three independent experiments. * *p* < 0.05, ** *p* < 0.01, *** *p* < 0.001 compared with LPS-treated group.

THMX was tested for its effects on the LPS-induced expression of pro-inflammatory factors in RAW 264.7 and BV2 cells. These cells were treated with 2.5–10 μM THMX for 2 h and then stimulated with LPS (0.5 μg/mL) for 24 h. Figure 2A–D demonstrates that in the LPS–induced RAW264.7 cells, THMX showed strong anti-inflammatory efficacy against NO release with an IC_50_ value of 5.77 ± 0.66 μM, as well as against PGE_2_, IL-6, and TNF-α release, with IC_50_ values of 9.70 ± 1.46, 13.34 ± 4.92, and 16.14 ± 2.19 μM, respectively. In addition, THMX significantly inhibited the LPS-induced production of NO, PGE_2_, IL-6, and TNF-α in the BV2 cells, with IC_50_ values of 11.93 ± 2.90, 7.53 ± 1.88, 10.87 ± 3.23, and 9.28 ± 0.40 μM, respectively (Figure 2E–H).

Dexamethasone, a well-known anti-inflammatory drug, was used as a positive control and has been examined for the regulatory effects of pro-inflammatory mediators in LPS-treated RAW264.7 and BV2 cells, obtaining IC_50_ values. Comparing the IC_50_ values of THMX and dexamethasone, it was confirmed that similar anti-inflammatory effects were observed (Table 1).

**Figure 3:**
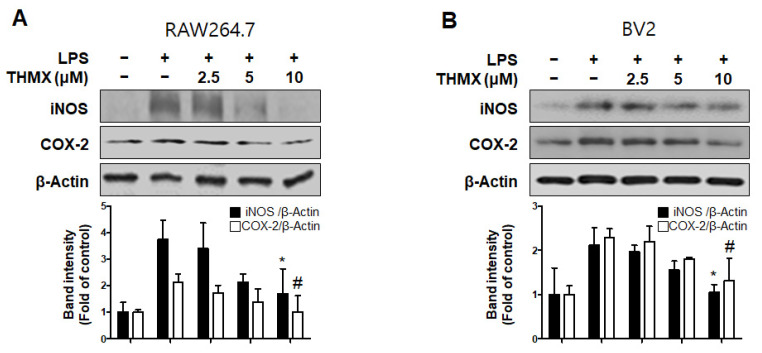
Protein expression levels of inducible nitric oxide synthase (iNOS) and cyclooxygenase-2 (COX-2) in lipopolysaccharide (LPS)-stimulated RAW 264.7 (**A**) and BV2 (**B**) cells. Cells were pretreated for 2 h with indicated concentrations of THMX and stimulated for 24 h with LPS (0.5 μg/mL). Representative blots from three independent experiments are shown. Immunoblots were quantified using ImageJ software (Version 1.53t). Band intensities were normalized to β-actin. * *p* < 0.05, # *p* < 0.05 compared with LPS-treated group.

Furthermore, an investigation of the inflammation-related protein expression revealed an increase in both the iNOS and COX-2 expression in LPS-induced cells, while the THMX pretreatment declined expression of iNOS and COX-2, as confirmed by Western blotting (Figure 3).

### 2.3. Effects of THMX on p65 Translation and MAPK Signaling Pathway in RAW 264.7 and BV2 Cells

**Figure 4:**
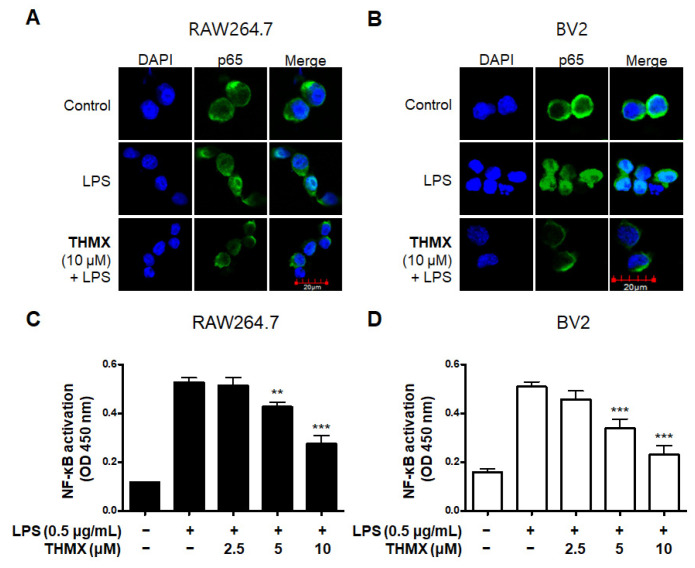
Effects of THMX on NF-κB (p65) localization (**A**,**B**) and DNA-binding activity (**C**,**D**) in RAW 264.7 and BV2 cells. Cells were pretreated with THMX for 2 h and stimulated with LPS (0.5 μg/mL) for 1 h. NF-κB localization (green) was visualized after immunofluorescent staining. Nuclei were stained with DAPI (blue). Commercially available NF-κB enzyme-linked immunosorbent assay (ELISA) kit was used as described in Materials and Methods. ** *p* < 0.01, *** *p* < 0.001 compared with LPS-treated group.

We used an immunofluorescence analysis to examine the expression of p65 NF-κB to further investigate THMX’s inhibitory effect on the production of pro-inflammatory factors in LPS-activated RAW 264.7 and BV2 cells. Compared with LPS-induced RAW 264.7 and BV2 cells (Figure 4A,B), THMX markedly reduced the LPS-mediated increase in the nuclear translocation of NF-κB (p65). Furthermore, the effects of THMX on the prevention of DNA-binding activity in the LPS-induced cells were also demonstrated (Figure 4C,D).

**Figure 5:**
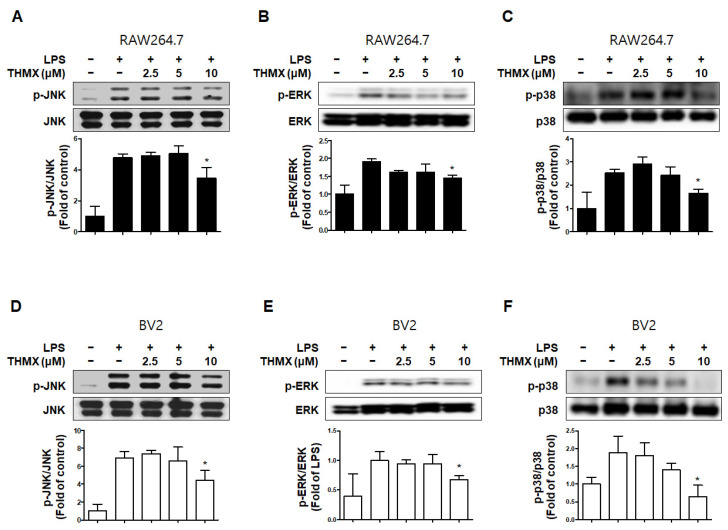
Effects of THMX on c-Jun N-terminal kinase (JNK) (**A**,**D**), extracellular signal-regulated kinase (ERK) (**B**,**E**), and p38 (**C**,**F**) phosphorylation in RAW 264.7 (A-C) and BV2 cells (D-F). Cells were pretreated with the indicated concentrations of THMX for 2 h and stimulated for 30 min with lipopolysaccharide (LPS, 0.5 μg/mL). Total abundance of each mitogen-activated protein kinase (MAPK) was used as a control. Representative blots from three independent experiments are shown. Immunoblots were quantified using ImageJ software (Version 1.53t). Band intensity was quantified normalized to each total form. * *p* < 0.05 compared with LPS-treated group.

In addition, we measured the levels of MAPK pathway proteins in the LPS- and/or THMX-treated cells. The RAW 264.7 and BV2 cells treated with LPS for 30 min exhibited increased p-JNK, p-p38, and p-ERK levels compared to the untreated control cells (Figure 5). In contrast, THMX significantly inhibited the expression of p-JNK, p-ERK, and p-p38 in the LPS-treated cells (Figure 5). As a result, THMX appears to inhibit LPS-induced neuroinflammatory cytokine production by suppressing the NF-κB and MAPK pathways.

### 2.4. Effects of THMX on HO-1 in RAW 264.7 and BV2 Cells

**Figure 6:**
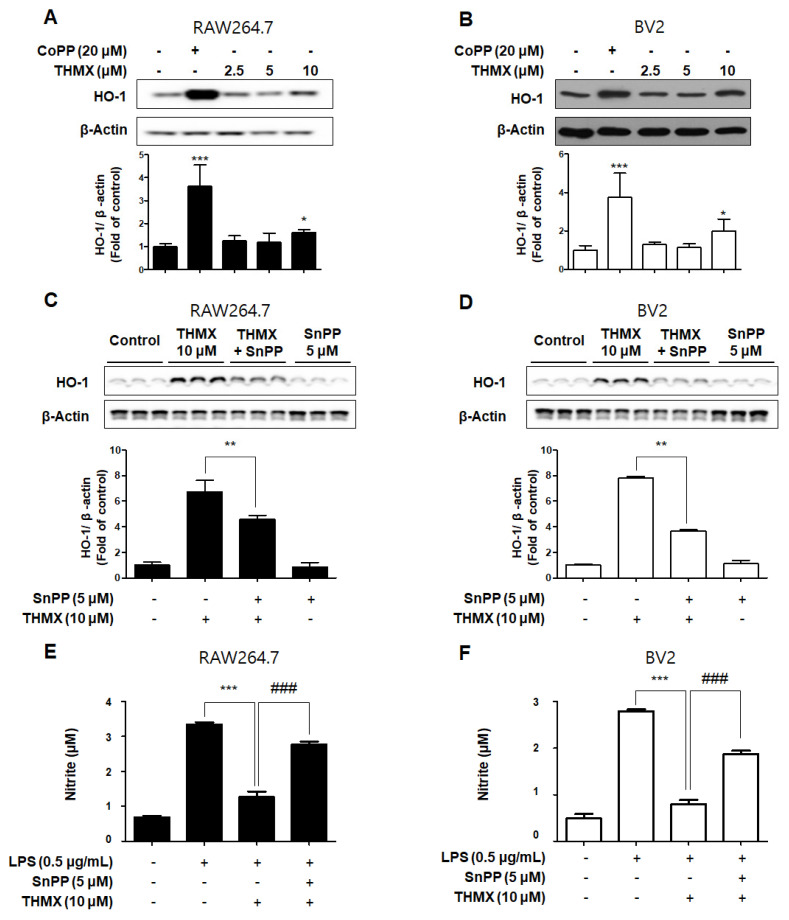
Effects of THMX on HO-1 in RAW 264.7 (**A**) and BV2 cells (**B**), and inhibitory effect of THMX on nitrite production through the regulation of HO-1 activity in RAW 264.7 (**C**) and BV2 (**D**) cells. (**A**,**B**): Cells were treated with THMX or CoPP (20 μM) for 12 h. Representative blots from three independent experiments are shown. Immunoblots were quantified using ImageJ software (Version 1.53t). Band intensity was quantified normalized to β-actin. * *p* < 0.05, *** *p* < 0.001 compared with control group. (**C**,**D**): Cells were treated with SnPP (5 μM) or THMX (10 μM) for 12 h. ** *p* < 0.01 compared with THMX treated group. (**E**,**F**): Cells were treated with 5 μM of SnPP or THMX and then stimulated for 24 h with lipopolysaccharide (LPS, 0.5 μg/mL). Data are presented as mean ± SD values of three independent experiments. *** *p* < 0.001 vs. LPS. ### *p* < 0.001 vs. LPS and THMX.

In our previous investigation, it was shown that THMX inhibits inflammation in RAW 264.7 and BV2 cells. The cells were exposed to the following THMX concentrations for 12 h before being tested by Western blotting in order to evaluate if THMX promoted the expression of HO-1. Cobalt protoporphyrin (CoPP) was selected as a kind of positive control. THMX enhanced the HO-1 expression in these cells considerably (Figure 6A,B). We included a set of experiments with tin protoporphyrin-IX (SnPP, a selective HO-1 activity inhibitor) in order to identify whether THMX’s neuroprotective and anti-inflammatory effects are related to HO-1 expression in RAW 264.7 and BV2 cells. First, SnPP significantly inhibited THMX-enhanced HO-1 protein expression (Figure 6C,D). Secondly, after 2 h of treatment with THMX (10 µM) with or without SnPP (5 µM), the cells were incubated for 24 h with LPS. THMX reduced the nitrite levels in the LPS-induced RAW 264.7 and BV2 cells (Figure 6E,F). The SnPP treatment, on the other hand, reversed THMX’s anti-inflammatory effects. SnPP alone had no effect on NO production after LPS stimulation, implying that THMX’s anti-inflammatory effects are controlled by HO-1 expression.

## 3. Discussion

In acute neuroinflammation, inflammatory mediators recover injured neurons and glial cells as a protective mechanism [29,30,31]. Chronic neuroinflammation, however, is more likely to result in increased neuronal deterioration [32,33,34]. Thus, therapeutic agents of inflammation have the potential to delay the onset of neurodegenerative diseases.

THMX (Figure 1A) is a xanthone that is abundantly found in Moraceae plants, including *Cudrania fruticosa* and *C. tricuspidate*, and is utilized as both a food and medicinal plant [18,35]. In the present study, two cell types (BV2 and RAW 264.7) were used to explore the inflammatory effects of THMX from *C. tricuspidata*. Higher concentrations of NO and PGE_2_ have neurotoxic effects, and LPS-activated microglia cause neuronal cell degeneration by producing pro-inflammatory cytokines and neurotoxic mediators, such as IL-6, TNF-α, iNOS, and COX-2 [36]. THMX pretreatment attenuates NO and PGE_2_ production, and THMX also lowered the LPS-induced expression of pro-inflammatory cytokines (IL-6 and TNF-α) and inhibited the protein expression of iNOS and COX-2 in the LPS-stimulated cells (Figure 2 and Figure 3).

NF-κB is a transcription factor that is indispensable for the expression of genes that control cellular functions, which include cell differentiation and inflammation [37]. It is released from the cytoplasm by the inhibitor protein IκBα and is moved into the nucleus. Translocated NF-κB interacts with the promoter region of the target genes, increasing the transcription of inflammatory cytokines, adhesion molecules, and chemokines [38]. In this research, we examined the mechanisms by which THMX inhibits neuroinflammation. The findings demonstrate that, as a result of controlling NF-κB activation, THMX had an inhibitory effect on the progress of p65 protein migration into the nucleus (Figure 4A,B). An analysis of the inhibitory protein IκBα expression demonstrated that pretreatment with THMX blocked LPS-induced phosphorylation and degradation, suggesting that THMX prevented NF-κB activation (Figure 4C,D).

One of the serine/threonine kinases that has the ability to send extracellular signals to the nucleus is MAPK. The synthesis of mediators carried via their signal transduction results in inflammatory reactions. The activation of transcription factors like NF-κB and activator protein (AP)-1, which are involved in the production of free radicals like NO and are necessary for the induction of early-stage inflammatory reactions, is caused by MAPK phosphorylation. [39]. In a concentration-dependent manner, pretreatment with THMX lowered the phosphorylation of JNK, ERK, and p38 MAPK by LPS in the RAW264.7 and BV2 cells (Figure 5). These findings imply that modulation of the MAPK signaling pathway is one of the most important mechanisms for THMX’s neuroinflammatory inhibitory effect.

Biliverdin, CO, and ferrous ions are generated when heme is degraded by HO. Both biliverdin and bilirubin have antioxidant and anti-inflammatory effects [40]. The anti-oxidative and anti-inflammatory effects by HO-1 expression regulate how LPS-induced iNOS is expressed in RAW264.7 and BV2 cells, which could prevent NO generation (Figure 6). According to our findings, THMX efficiently stimulated expression of HO-1 in the RAW 264.7 and BV2 cells.

For the development of functional food products utilizing the anti-inflammatory and cell-protective effects of THMX, it is required to examine several essential evaluations, such as bioavailability, metabolism, and safety. In addition, further investigation should be focused on optimization extractions and the isolation of THMX from *C. tricuspidata* or the chemical synthesis of THMX. On the other hand, our previous research confirmed the presence of THMX in *C. tricuspidata* extracts through an HPLC analysis. Therefore, we plan to obtain quantitative data on the content of THMX in *C. tricuspidata* through an HPLC analysis after obtaining a large amount of THMX in further research. Nevertheless, while this study has several limitations, the primary focus of our research is the first-time confirmation of the anti-inflammatory mechanism of THMX in RAW264.7 and BV2 cells. This study serves as a foundational exploration, indicating the potential utility of THMX as a future anti-inflammatory material for the development of functional food and pharmaceutical products.

In summary, the results of this study strongly suggest that THMX significantly suppressed the activation of two cell types (BV2 and RAW 264.7). THMX not only reduced MAPK activation, but also inhibited NF-ĸB activation and HO-1 signaling. This suggests that THMX from *C. tricuspidata* is a potential candidate for the development of functional food material with an anti-inflammatory effect.

## 4. Materials and Methods

### 4.1. Materials

Roswell Park Memorial Institute 1640 (RPMI1640) and fetal bovine serum were among the tissue culture materials offered by Gibco BRL (Grand Island, NY, USA). The company Sigma-Aldrich (St. Louis, MO, USA) provided all of the reagents. Anti-iNOS (catalog no. sc-7271), anti-COX-2 (catalog no. sc-376861), anti-p65 (catalog no. sc-8008), anti-HO-1 (catalog no. sc-390991), and anti-actin (catalog no. sc-47778) primary antibodies were available from Santa Cruz Biotechnology (Santa Cruz, CA, USA), in addition to anti-p-JNK (catalog no. #9251), anti-p-ERK (catalog no. #9201), anti-p-p38 (catalog no. #9211), anti-JNK (catalog no. #9252), anti-p-ERK (catalog no. #9202), and anti-p38 (catalog no. #9212) from Cell Signaling Technology (Danvers, MA, USA); and anti-mouse (catalog no. ap124p) and anti-rabbit (catalog no. ap132p) secondary antibodies from Millipore (Billerica MA, USA). R&D Systems (Minneapolis, MN, USA) offered enzyme-linked immunosorbent assay (ELISA) kits for measuring PGE_2_ (catalog no. #KGE004B), IL-6 (catalog no. #M6000B), and TNF-α (catalog no. #MTA00B). THMX was isolated from *C. tricuspidata* by a previously described method, and the structure of the compound was revealed through the analysis of several spectroscopic data, which include nuclear magnetic resonance and mass spectroscopy data, and it was in agreement with a previous report [18].

THMX: Yellow needles (CH_3_OH), mp 182 –185 °C; UV $\lambda_{\max}^{\mathrm{MeOH}}$ nm (log ε): 235 (4.05), 261 (4.14), 316 (3.77), 376 (3.78); + NaOAc: 262, 390; IR (KBr) ν_max_ cm^−1^: 3330, 1645, 1620, 1580, 1460, 1200, 820; EI-MS *m*/*z*: 342 [M^+^, 65], 327 [100], 316 [8], 300 [15], 257 [8], 190 [2], 69 [5]; HREI-MS m/z 342.1113 (calcd for C_19_H_18_O_6_, 342.1103); ^1^H NMR and ^13^C NMR data. From a comparison with ^1^H- and ^13^C-NMR data in the literature, the structure of THMX was determined to be 1,6,7-Trihydroxy-2-(1,1-dimethyl-2-propenyl)-3-methoxyxanthone [35].

### 4.2. Cell Culture and Viability Assays

RAW264.7 and BV2 cells were obtained from Professor Yun-Cheol Kim (Wonkwang University). The method previously described was used to incubate the RAW264.7 and BV2 cells. At 37 °C in a 95% air environment and humidified 5% CO_2_, the cells were maintained at a density of 5 × 10^5^ cells/mL in RPMI1640 containing 10% heat-inactivated fetal bovine serum (FBS) and 1% antibiotics (penicillin–streptomycin) [41].

### 4.3. Measurement of Nitrite Oxide (NO) Generation

The production of nitrite, a persistent byproduct of NO oxidation, was examined as an indication of NO generation in the cells. In brief, the nitrite concentration in the conditioned media was identified using a method based on the Griess reaction [42]. The details of this assay have been described previously [41].

### 4.4. PGE_2_ Assay

The PGE_2_ level of each sample was determined with a commercial kit from R&D Systems (Minneapolis, MN, USA) using the previously referred-to method [41].

### 4.5. Assays for IL-6 and TNF-α

The IL-6 and TNF-α levels were evaluated in each sample’s culture medium using commercially available kits (BioLegend, San Diego, CA, USA). The assay was conducted as directed by the instructions of manufacturers. In brief, the cells were seeded in 48-well culture plates at a density of 2 × 10^5^ cells/well. The supernatant was taken after incubation and used in cytokine ELISA kits to assess the levels of IL-6 and TNF-α.

### 4.6. Western Blotting Analysis

The cells were pelleted and rinsed with phosphate-buffered saline (PBS) before being lysed in RIPA buffer. Using a protein assay dye reagent concentrate from Bio-Rad Laboratories (#5000006; Hercules, CA, USA), equal amounts of protein (30 μg) were measured, combined with sample loading buffer, and separated by SDS-PAGE (7.5%: iNOS(1:1000), COX-2(1:1000); 12%: JNK(1:2000), ERK(1:2000), p38(1:2000), HO-1(1:1000)). The proteins were isolated and put to nitrocellulose membranes. By incubation with skim milk, nonspecific binding to the membrane was prevented. The membrane was coated with primary antibodies overnight at 4 °C before being incubated with horseradish peroxidase-conjugated secondary antibodies for 1 h (1:5000, room temperature). The bands were detected via ECL Western blotting substrate (cat. no. 34095, ThermoFisher scientific, Waltham, MA, USA). The bands were quantified by densitometry and normalized to β-actin (1:1000) or each total form (Image J, National Institutes of Health, Bethesda, MD, USA). 

### 4.7. NF-κB Localization and Immunofluorescence

The cells were maintained on Lab-Tek II chamber slides, treated with THMX (10 μM) for 2 h, and then triggered with LPS (0.5 μg/mL) for 1 h for analysis of the location of NF-κB. Following that, the cells were permeabilized with cold acetone and fixed in formalin. The cells were probed with an anti-p65 antibody (catalog no. sc-8008; 1:200, Santa Cruz Biotechnology, Dallas, TX, USA, 37 °C, 1 h) before being incubated with a secondary antibody (catalog no. A-11001; 1:1000, Invitrogen, 37 °C, 1 h) labeled with fluorescein isothiocyanate (Alexa Fluor 488). The cells were incubated for 30 min with DAPI (1 μg/mL), rinsed with PBS for 5 min, and then treated with VectaShield (50 μL) (Vector Laboratories, Burlingame, CA, USA) for 30 min to visualize the nuclei. A Zeiss fluorescence microscope was used to observe and photograph the stained cells (Provis AX70; Olympus Optical, Tokyo, Japan) [43].

### 4.8. Measurement of DNA-Binding Activity of NF-κB

NF-κB DNA binding activity was performed using the nuclear extracts of the cells. The THMX was treated at a concentration of 2.5–10 μM, and cytoplasmic and nuclear fractions were obtained from an inflammatory reaction that occurred cell using a nuclear extraction kit (Cayman, Ann Arbor, MI, USA). They were then analyzed at a wavelength of 450 nm using the NF-κB transcription factor assay kit (Cayman, Ann Arbor, MI, USA).

### 4.9. Statistical Analysis

All of the data are shown as the mean ± standard deviation of three independent experiments. To compare the three groups, a one-way analysis of variance was used, followed by Tukey’s multiple comparison tests. The GraphPad Prism program, version 5.01, was used for the statistical analyses (GraphPad Software, San Diego, CA, USA). * *p* < 0.05, ** *p* < 0.01, *** *p* < 0.001, # *p* < 0.05, and ## *p* < 0.001 was considered to indicate a statistically significant difference.

## 5. Conclusion

We have confirmed that THMX isolated from *Cudrania tricuspidata* exerted anti-inflammatory effects in LPS-stimulated RAW24.7 and BV2 cells. These inhibitory effects were identified by decreasing the production of pro-inflammatory mediators, such as NO, PGE_2_, IL-6, and TNF-α, and reducing the protein expression levels of iNOS and COX-2. Additionally, this compound showed anti-inflammatory effects via the induction of HO-1 expression and the inactivation of the NF-κB and MAPK signaling pathways. Our findings suggest that *C. tricuspidata*, which contains THMX, could be a good candidate for the development of functional food as an anti-inflammatory regulator.

## Figures and Tables

**Table 1:** IC_50_ value of THMX on the pro-inflammatory mediators in lipopolysaccharide (LPS)-stimulated RAW264.7 and BV2 cells.

Pro-Inflammatory Mediators	Name	RAW264.7 (μM)	BV2 (μM)
NO (IC_50_)	THMX	5.77 ± 0.66	11.93 ± 2.90
	Dexamethasone ^a^	5.38 ± 1.43	7.14 ± 2.16
PGE_2_ (IC_50_)	THMX	9.70 ± 1.46	7.53 ± 1.88
	Dexamethasone ^a^	7.06 ± 0.88	4.41 ± 0.59
IL-6 (IC_50_)	THMX	13.34 ± 4.92	10.87 ± 3.23
	Dexamethasone ^a^	7.02 ± 0.78	8.03 ± 0.65
TNF-α (IC_50_)	THMX	16.14 ± 2.19	9.28 ± 0.40
	Dexamethasone ^a^	9.31 ± 0.45	8.53 ± 0.93

^a^ Positive control.

## Data Availability

Not applicable.

## Sample Availability

Samples of the compounds are not available from the authors.
